# IL-23/IL-17 Axis Activates IL-1β-Associated Inflammasome in Macrophages and Generates an Auto-Inflammatory Response in a Subgroup of Patients With Bullous Pemphigoid

**DOI:** 10.3389/fimmu.2019.01972

**Published:** 2019-08-27

**Authors:** Sébastien Le Jan, Céline Muller, Julie Plee, Anne Durlach, Philippe Bernard, Frank Antonicelli

**Affiliations:** ^1^Laboratory of Dermatology, EA7509 IRMAIC, University of Reims-Champagne-Ardenne, Reims, France; ^2^Department of Dermatology, University Hospital, University of Reims-Champagne-Ardenne, Reims, France; ^3^Laboratory Pol Bouin, Hospital Maison Blanche, University Hospital, University of Reims-Champagne-Ardenne, Reims, France; ^4^Department of Biological Sciences, Immunology, UFR Odontology, University of Reims-Champagne-Ardenne, Reims, France

**Keywords:** bullous pemphigoid, auto-inflammation, inflammasome, macrophages, IL-1β, IL-17

## Abstract

Bullous Pemphigoid (BP) is a skin autoimmune blistering disease characterized by immune-mediated degradation of the dermo-epidermal junction and release of a large number of inflammatory cytokines. Interleukin-1β (IL-1β) is a pleiotropic pro-inflammatory cytokine associated with inflammasome activation and known to be pivotal in several auto-immune and auto-inflammatory diseases. We sought to clarify the presence of inflammasome-dependent IL-1β and to investigate its role in BP. Skin biopsy specimens (*n* = 13), serum (*n* = 60), blister fluid (*n* = 26), and primary inflammatory cells from patients with BP were used to investigate inflammasome activation and function. We here highlighted a differential occurrence of a functional *in situ* inflammasome in patients with BP, biologically distinguished by IL-1β and NLRP3 expression. Clinically, elevated IL-1β levels were associated with the presence of erythema and urticarial plaques reflecting the inflammatory phase preceding blister formation. We further identified IL-17 and IL-23 as important molecules favoring IL-1β expression in monocyte-derived macrophages from BP patients. Finally, we demonstrated the ability of IL-1β to stimulate the release of the matrix metalloproteinase-9 in those macrophages, reinforcing the role of IL-1β in the auto-amplification loop of the inflammatory response associated to BP. However, whether this inflammasome is an epiphenomenon associated with BP disease or constitutes an amplification inflammatory step in certain patients still need to be determined. In the context of a precision medicine approach, our findings allowed us to delineate a subgroup of patients with BP that showed similarities with auto-inflammatory diseases. Subsequently, this opens up alternative therapeutic strategies targeting IL-1β pathway in the aim to control the early, pre-blistering inflammatory phase. Ultimately, this could also help in reducing the detrimental effects associated with high doses of corticosteroids treatment.

## Introduction

Bullous pemphigoid (BP) is the most common auto-immune blistering skin disease, clinically characterized by urticarial erythema with tense blisters, and immunologically distinguished by the presence of autoantibodies directed against two structural proteins of the dermal-epidermal junction (DEJ), BP180, and BP230. Disease progression is related to a local autoantibody-induced inflammatory response that can be characteristically subdivided into an early non-bullous urticarial/erythematous phase and a later lesional phase with blisters and erosions (1). Although it has been shown that persistent elevated titer of anti-BP180 ELISA autoantibody was a good indicator of further relapse of BP (2), we also demonstrated that variation in the inflammatory response could affect both disease activity and relapse occurrence (3–6). We here further investigated whether self-directed inflammation driven *via* activation of innate immune pathways could be related to the auto-inflammation concept, and therefore to the expression of a functional *in situ* inflammasome in BP.

The “sterile” NLRP3 inflammasome has been increasingly involved in non-infectious inflammatory responses related to autoimmune diseases (7), leading to the production of bioactive IL-1β. Indeed, IL-1ß appears to be pivotal in connecting the innate immune response and the adaptive immune responses, particularly by driving lymphocyte polarization (8–11). “Sterile,” i.e., non-infectious inflammation originates from endogenous factors including extracellular matrix (ECM) components released from tissue damage, which act as danger-associated molecular patterns (DAMPs) (12). In BP, we previously showed that elastase released in skin blisters induced BP180 fragmentation (13), and the presence of biological active peptide within the blister fluid (BF) (4). Those latter peptides were chemoattractant for neutrophils and favored the release of elastase, therefore creating an auto feedback loop (4). However, IL-1β reported concentrations in BF remain controversial (14–16) and could not be explained by IL-1β gene polymorphism in BP disease (17). Then, studies are still needed to define the molecular mechanisms leading to NLRP3 inflammasome activation and to the expression and activation of IL-1β, notably with respect to BP disease activity.

Activation of the NLRP3 inflammasome requires 2 signals: a priming signal that involves transcriptional upregulation of NLRP3 and of pro-inflammatory cytokines such as IL-1β; and an activating step that can be induced by various triggers leading to pro-caspase 1 expression and activation, and ultimately to IL-1β processing into its active form (18). Besides DAMPs, other inflammatory molecules classically associated with BP pathophysiological mechanisms, such as pro-inflammatory cytokines, complement, and reactive oxygen species (ROS) were shown to prime the NLRP3 inflammasome (19, 20). Notably, recent studies showed the implication of IL-17 in NLRP3 priming, IL-1ß release and activation through NF-kB and ROS pathways, respectively (21, 22). Besides, we previously demonstrated the presence of IL-17 in the serum of BP patients, but no direct correlation could be drawn with the extent of the disease (5). Additionally, BP autoantibodies could also participate to NLRP3 inflammasome activation, as demonstrated in monocyte/macrophages from SLE (23).

In this study we investigated in BP the concept of the inflammasome associated auto-inflammatory diseases being linked to the expression and activation of IL-1β. To that end, we measured the expression of several markers of inflammasome component, especially IL-1β and NLRP3, at the site of skin lesion and in biological fluids (serum and BFs) from patients with BP. We further assessed IL-1β clinical involvement by evaluating correlations between IL-1β levels and clinical activity of patients assessed by their BPDAI (bullous pemphigoid disease activity index). We then delineated the role of BF in regulating the auto-inflammatory response associated to BP through the involvement of several of its components including IL-17 and IL-23, as well as BP autoantibodies in NLRP3 inflammasome priming and activation. We finally evaluated the role of IL-1β in the amplification of the inflammatory response in BP.

## Materials and Methods

### Study Design and Patients

This ancillary study is part of a main prospective, single-center study that was conducted in the Department of Dermatology at Reims University Hospital (French Referral Center for Autoimmune Bullous Diseases) between September 2013 and July 2017. Seventy-one consecutive patients with newly diagnosed BP were included in this ancillary study on the basis of clinical features typical of BP with the presence of at least 3 of 4 criteria by Vaillant et al. (24), subepidermal blistering on skin biopsy, and deposits of IgG, C3, or both in a linear pattern along the epidermal basement membrane zone by direct immunofluorescence (sex ratio F/H: 1,45; mean age, 81.7 years old; range, 55–98 years old). For the patients included, serum, BF and a skin biopsy specimen were collected at time of diagnosis, when possible. Clinical activity of the disease was evaluated for each patient using BPDAI score (25). The BPDAI measures separate scores for mucous membrane and skin activities, the latter evaluating separately both cutaneous urticarial/erythema (non-bullous phase) and cutaneous blisters/erosions (blistering phase). Serum samples used as controls were harvested from age- and sex-matched patients that were admitted to the Trauma Department of Reims University Hospital and that showed neither biological inflammatory syndrome (C-reactive protein levels lower than 10 mg/l) nor autoimmune diseases.

### Ethics Statement

The investigation was conducted under the approval of the Ethic Committee of the University Hospital of Reims (CNIL authorization DR-2013-320), and all of the subjects gave their informed and written consent before participating in the study in accordance with the Helsinki Declaration.

### Autoantibody and Cytokine Level Analysis

Anti-BP180 and anti-BP230 autoantibodies as well as IL-1β levels were measured in control and BP sera and in BF using specific commercially available enzyme-linked immunosorbent assays (ELISAs) according to the manufacturer's instructions (MBL, Nagoya, Japan and Ebiosciences, Paris, France, respectively). IL-1β levels were also detected in supernatants issued from THP-1- and BP monocyte-derived macrophages. Finally, IL-17 and IL-23 levels were analyzed in BF using U-PLEX assay (MesoScale Diagnostics, Rockville, USA). U-PLEX technology allows multiplex cytokine measurement and is based on electro-chemiluminescence detection. Briefly biotinylated capture anti-IL17 and anti-IL-23 antibodies were coupled to U-PLEX Linkers. The U-PLEX Linkers then self-assembled onto unique spots on the U-PLEX plate. After binding to the capture antibodies, detection antibodies conjugated with electro-chemiluminescent labels (MSD GOLD SULFO-TAG) bound to the analytes to complete the sandwich immunoassay. The plate was then placed into an MSD instrument where the amounts of IL-17 and IL-23 present in BF were measured.

### Cell Isolation and Cell Culture

The THP-1 (Tohoku Hospital Pediatrics-1) cell line, derived from the peripheral blood of a 1 year old human male with acute monocytic leukemia (26) and kindly provided by Dr S. Hart (University of Edinburgh, Scotland, UK), was cultured in RPMI 1640 medium (Life Technologies, NY, USA) supplemented with 10% FBS, 2 mmol/L glutamine, 25 U/mL penicillin, and 25 U/mL streptomycin at 37°C in a humidified 5% CO_2_ incubator. For macrophage differentiation, THP-1 cells were incubated with phorbol myristate acetate (PMA) 50 nM for 68 h.

Peripheral Blood Mononuclear Cells (PBMCs) and polymorphonuclear (PMN) cells from control subjects and patients with BP were isolated using a density gradient technique from EDTA-treated whole blood (GranuloSep; Eurobio-Abcys, Courtaboeuf, France). Monocytes were then positively selected and purified from PBMCs by using CD14 immuno-magnetic beads according to the manufacturer's instructions (MACS; Miltenyi Biotec, Bergisch Gladbach, Germany). All leukocytes were cultured in RPMI 1640 medium (Life Technologies, NY, USA), 2 mmol/L glutamine, 25 U/mL penicillin, and 25 U/mL streptomycin at 378°C in a humidified 5% CO_2_ incubator. Monocytes were differentiated into macrophages by culturing them for 7 days in the presence of autologous serum (10%).

### *In vitro* Inflammasome Activation Assay

In order to analyze the priming of the NLRP3 inflammasome, THP-1- and BP monocyte-derived macrophages were stimulated with either lipopolysaccharide (LPS) (1 μg/ml) (Santa Cruz Biotechnology, Dallas, USA) or BF 25% for 3 h. Priming was also induced by recombinant IL-17 or recombinant IL-23 (Bio-Techne, Lille, France) in BP monocyte-derived macrophages. For IL-17 stimulation, a concentration of 1 ng/ml was chosen to be closer to the maximal BF concentration that was measured while staying in the range of the ED_50_ (3–15 ng/ml). While ED_50_ for recombinant human IL-23 was around 0.3 ng/ml, the concentration of IL-23 used for stimulation was also 1 ng/ml to remain in the same range of concentrations than the ones used for IL-17. Cells were then harvested for NLRP3 and IL-1β gene expression analysis. Activation of the inflammasome was induced by BF 25% for 30 min in either LPS-primed THP-1- and BP monocyte-derived macrophages or LPS-primed PMN cells. Supernatant were then collected for IL-1β concentration measurement. Nigericin 20 μM (Santa Cruz Biotechnology, Dallas, USA), by acting as a potassium ionophore, was used as a positive control of inflammasome activation. Added together with either Nigericin or BF to the cells, Glibenclamide 50 μM (Santa Cruz Biotechnology, Dallas, USA) was employed as an inhibitor of inflammasome activation.

### Gene Expression Analysis

Total RNA from THP-1- and BP monocyte-derived macrophages was extracted with TRI Reagent (Euromedex, Souffelweyersheim, France). Reverse transcription was performed from 1 μg of total RNA by using the Maxima First Strand cDNA kit with dsDNAse (ThermoFisher Scientific, Waltham, USA), according to the manufacturer's instructions. *IL-1*β (Forward primer: 5′GGATATGGAGCAACAAGTGG 3′; Reverse primer: 5′ATGTACCAGTTGGGGAACTG 3′) and *NLRP3* (Forward primer: 5′CTTCTCTGATGAGGCCCAAG 3′; Reverse primer: 5′GCAGCAAACTGGAAAGGAAG 3') gene expression was analyzed by using real-time PCR with Power SYBR Green PCR Master mix (ThermoFisher Scientific, Waltham, USA) on the Stratagene Mx3005P system (Agilent Technologies, Santa Clara, USA). Relative quantification was performed with *GAPDH* as a reference gene.

### Immunohistochemistry

Perilesional skin biopsy specimens from 13 patients with BP before the start of treatment were obtained. Tissues were fixed in paraformaldehyde, embedded in paraffin, and sectioned. Immunostaining with a mouse monoclonal anti-human NLRP3 antibody (clone Nalpy3-b, Enzo Life Sciences, Villeurbanne, France) or a mouse monoclonal anti-human CD163 antibody (Clone MRQ26, Roche Diagnostics, Meylan, France) was performed using routine methods with a biotinylated anti-mouse secondary antibody (BA-2000; Vector Laboratories, Burlingame, USA) and the ABC-peroxidase complex (Vector Laboratories, Burlingame, USA) with diaminobenzidine-H_2_O_2_ used as the chromogen for detection. In order to identify cells positive for both NLRP3 and CD163, immunohistochemistry for both markers was performed on 4 μm-thick serial BP skin section. Virtual slide images for NLRP3 and CD163 staining were taken using the VS120 Virtual Slide Microscope (Olympus France, Rungis, France). Virtual slide images corresponding to NLRP3 and CD163 staining were opened at the same time with OlyVIA software (Olympus France, Rungis, France) and synchronized for analysis.

### Statistical Analysis

Descriptive statistics such as means and SEMs were conducted for all quantitative measures. The distribution of the variables was assessed using D'Agostino and Pearson omnibus normality test. As population could not be assumed to be normal and some of the groups examined were small, we used non-parametric testing to compare populations in this study. Comparisons between two groups were performed using the exact Wilcoxon signed-rank test for paired data and the Mann–Whitney test for unpaired data. Correlations were performed using non-parametric Spearman's correlation test. The results were considered significant if *p-*values were 0.05 or less.

## Results

### Presence of IL-1β-Related Inflammasome in a Subgroup of Patients With Severe BP

**Figure 1:**
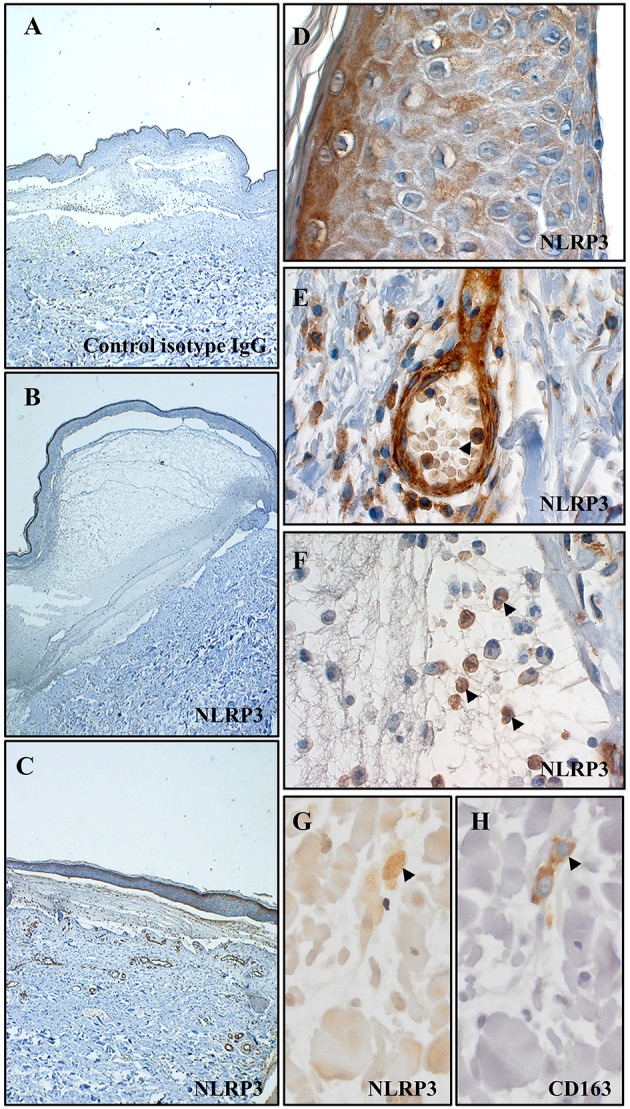
NLRP3 was expressed at site of lesion in BP. Immunohistochemistry for NLRP3 was performed on skin biopsy specimen from 13 patients with BP. Representative pictures of technical negative control (with control isotype IgG), of BP skin negative for NLRP3 and of BP skin positive for NLRP3 were shown in **(A–C)** respectively (magnification x40). More precisely, immunohistochemistry for NLRP3 was analyzed in the epidermis **(D)**, in the dermis **(E)** and in the blister cavity **(F)** of BP skin. To note, arrowheads indicated polymorphonuclear cells in the lumen of a blood vessel in **(E)** and in the blister cavity in **(F)**. Serial staining for NLRP3 **(G)** and CD163 **(H)** was achieved in dermis and NLRP3-positive CD163^+^ macrophage was indicated by an arrowhead (magnification x400).

We first looked for the presence of an *in situ* inflammasome in BP through the detection of NLRP3 by immunohistochemistry (IHC) on 13 BP skin biopsy specimens collected at baseline (Figures 1A–H). NLRP3 was highly expressed in 6/13 patients with BP (Figure 1C), slightly in 4/13 patients, and not detected in 3/13 patients (Figure 1B). When detected, NLRP3 was expressed by keratinocytes in the epidermis (Figure 1D). We further showed NLRP3 expression in the dermis and in the blister cavity, especially in skin resident cells (endothelial cells, fibroblasts; Figure 1E) and infiltrated inflammatory cells including PMN cells (Figure 1F) and macrophages (Figure 1G). Noteworthy, PMN cells also expressed NLRP3 in the lumen of the blood vessel at proximity of the lesion site (arrowhead in Figure 1E). Macrophages were further characterized by IHC performed on serial slides of BP skin biopsy specimen, which identified NLRP3-positive CD163^+^ macrophage (Figures 1G,H) among the innate immune cell population observed at the vicinity of the blister.

**Figure 2:**
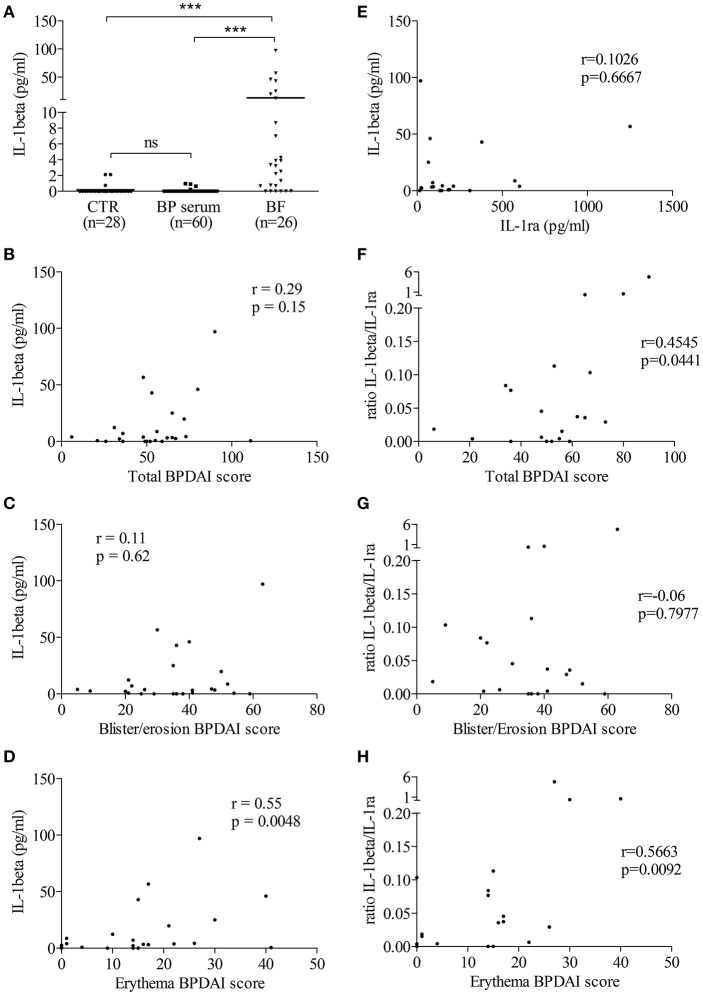
*In situ* IL-1β levels were related to BP disease activity. **(A)** IL-1β levels were measured in biological fluids of control and BP patients using ELISA (Mann–Whitney test was used to compare populations; ns, not significant and ***p < 0.001). Correlations between total **(B,F)**, blister/erosion **(C,G)**, and erythema **(D,H)** BPDAI scores and both IL-1β **(B–D)** and the IL-1β/IL-1ra ratio, used as inflammatory marker **(F–H)**, were analyzed using the non-parametric Spearman's correlation test. **(E)** Levels of IL-1ra, the natural antagonist of IL-1β, were measured in BF of patients with BP and correlation between IL-1β and IL-1ra was analyzed using the non-parametric Spearman's correlation test.

Then, we determined the level of IL-1β in biological fluids of patients with BP and of control subjects. IL-1β was not detected or found at very low levels both in sera from patients with BP (*n* = 60; mean: 0.046 ± 0.024 pg/mL) and in sera from control subjects (*n* = 28; mean: 0.178 ± 0.107 pg/mL; Figure 2A). In contrast to BP sera, IL-1β levels were significantly enhanced in BF of patients with BP (*n* = 26; mean: 13.17 ± 4.553 pg/ml; *p* < 0.001; Figure 2A). However, IL-1β expression was heterogeneously distributed with 6 out of the 26 BF in which IL-1β was not detected, while IL-1β concentrations could raise up to 100 pg/ml in other BFs (Figure 2A). IL-1β concentration in the BF was correlated with the skin erythema/urticaria activity (*n* = 25; *r* = 0.55; *p* = 0.0048; Figure 2D), but neither with the total BPDAI score (*n* = 25; *r* = 0.29; *p* = 0.15; Figure 2B), nor with the skin BPDAI subscore associated with blisters and erosions (*n* = 25; *r* = 0.11; *p* = 0.62; Figure 2C). In order to further demonstrate the biological activity of IL-1β, we also measured the expression of its natural receptor antagonist IL-1ra, which works as a decoy receptor to limit IL-1β –associated inflammatory process. As IL-1β secretion was not correlated to IL-1ra production in BF of patients with BP (Figure 2E), we analyzed the ratio IL-1β vs. IL-1ra. The IL-1β/IL-1ra inflammatory ratio was associated with disease activity (Figures 2F–H). Indeed, the IL-1β /IL-1ra ratio was significantly correlated both with the total BPDAI score and with the erythema/urticaria BPDAI subscore (Figures 2F–H, *n* = 20; *r* = 0.4545, *p* = 0.0441 and *r* = 0.5663, *p* = 0.0092, respectively), but not with the blisters/erosions BPDAI subscore (Figure 2G, *n* = 20, *r* = 0.06, *p* = 0.7977).

### BF Induced “Priming” and Activation of Inflammasome in Macrophages

**Figure 3:**
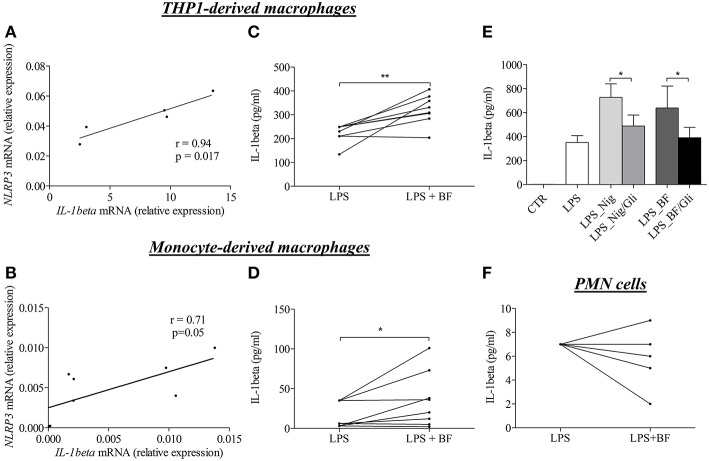
BF of BP patients had the ability to induce “priming” and activation of NLRP3 inflammasome in macrophages. Using quantitative RT-PCR, NLRP3, and IL-1β mRNA relative expression were analyzed in both THP-derived macrophages **(A)** and in BP serum-induced CD14+ monocyte-derived macrophages **(B)** stimulated for 3 h with BF. Correlation between IL-1 and NLRP3 was then investigated using the non-parametric Spearman's correlation test. Activation of inflammasome was examined through IL-1β detection by ELISA in both THP-derived macrophages **(C)** and in serum-induced CD14+ monocyte-derived macrophages **(D)** primed with LPS for 3 h and stimulated with BF for 30 min (*n* = 8; Wilcoxon's test for paired data was applied for statistical analysis; *p < 0.05 and **p < 0.01). The effect of glibenclamide, a specific inhibitor of NLRP3 inflammasome, was studied on nigericin or BF-induced IL-1β secretion in LPS-primed THP-1-derived macrophages **(E)** (*n* = 7; Data are shown as the mean ± SEM; Wilcoxon's test for paired data was applied; *p < 0.05). Activation of inflammasome by BF was also examined in LPS-primed BP PMN cells through IL-1β detection by ELISA **(F)**.

To investigate whether enhanced IL-1β concentrations were related to the NLRP3 inflammasome activation, we then investigated whether the BF itself could favor the expression of IL-1β in macrophages. For this purpose, we first evaluated the effects of BF on the “priming” step of the inflammasome induction by measuring both NLRP3 and IL-1β mRNA expression in 2 different models of macrophages *in vitro*. In both THP-1- and serum-induced CD14^+^ monocyte-derived macrophages stimulated with BF for 3 h, the increase of NLRP3 mRNA was associated with an increased expression of the IL-1β mRNA as demonstrated by a significant positive correlation between IL-1β and NLRP3 mRNA expressions under BF stimulation (*r* = 0.94, *p* = 0.017 and *r* = 0.71, *p* = 0.05, respectively; Figures 3A,B).

Following the investigation of the “priming” step, we investigated whether factors within the BF could also participate to the “activation” step of the inflammasome characterized by the production of active IL-1β upon CASPASE-1 activation. In both THP-1- and monocyte-derived macrophages models, BF increased IL-1β secretion in LPS-primed macrophages (Figures 3C,D). Such activation was further confirmed by use of nigericin, a specific inducer of inflammasome activation, and glibenclamide a selective inhibitor of NLRP3 inflammasome activation, respectively. Indeed, both nigericin- and BF-induced IL-1β secretion was significantly inhibited by glibenclamide in THP-1-derived macrophages (Figure 3E). In contrast, BF did not increase IL-1β secretion in PMN cells originated from BP patients and primed with LPS for 3 h before BF stimulation (Figure 3F).

### IL-17 and IL-23 Had the Ability to Prime IL-1β-Related Inflammasome in BP Macrophages

**Figure 4:**
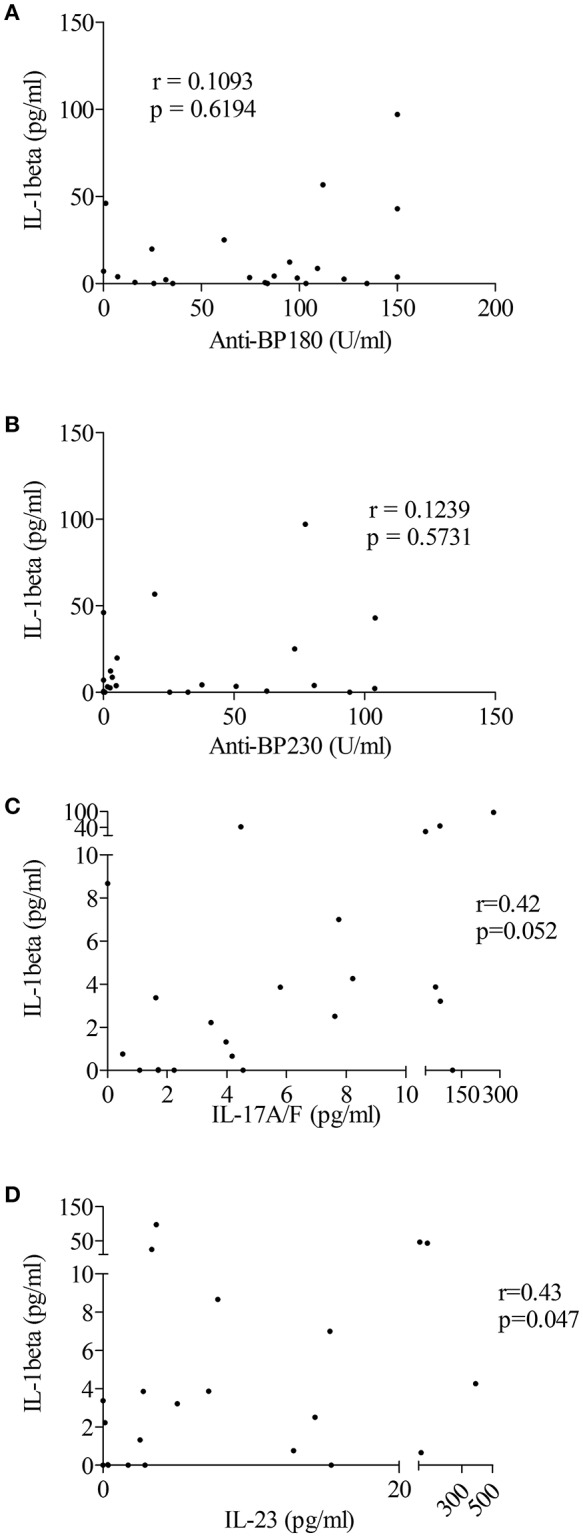
IL-1β levels were associated with pro-inflammatory cytokines but not autoantibodies in BF of patients with BP. Levels of anti-BP180 **(A)** and anti-BP230 **(B)** autoantibodies but also of the pro-inflammatory cytokines IL-17 **(C)** and IL-23 **(D)** were measured in BF of BP patients using ELISA before testing any correlation with IL-1β levels (non-parametric Spearman's correlation test was used for statistical analysis with *n* = 23 for autoantibodies and *n* = 22 for cytokines).

Based on recent studies showing the ability of autoantibodies and cytokines such as IL-17 to induce “priming” and activation of the inflammasome, we sought correlation between immunological and biological data, such as the levels of anti-BP180 and anti-BP230 autoantibodies or pro-inflammatory cytokines IL-17 and IL-23, and levels of IL-1β in BF of patients with BP (Figures 4A–D). A positive and significant correlation was established between BF levels of IL-1β and the concentrations of both IL-17 and IL-23 in BF of patients with BP (*n* = 22; *r* = 0.42, *p* = 0.05 and *r* = 0.43, *p* = 0.05, respectively; Figures 4C,D).

**Figure 5:**
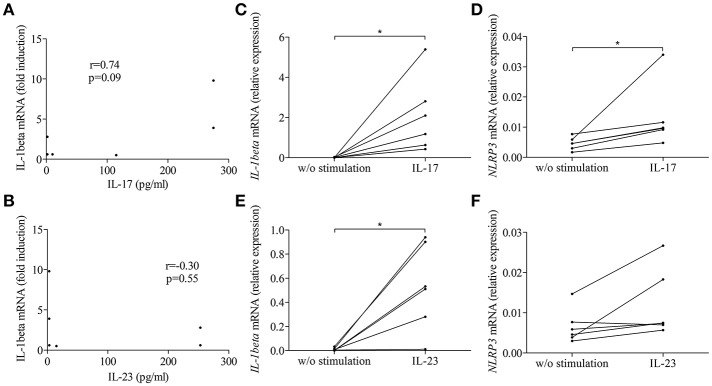
IL-17 and IL-23 induced IL-1β expression in BP monocyte-derived macrophages. Correlations were tested between macrophage-expressed IL-1β and the concentrations of IL-17 **(A)** and IL-23 **(B)** measured in BFs used to prime BP monocyte-derived macrophages. IL-1β **(C,E)** and NLRP3 **(D,F)** mRNA expressions were investigated in CD14+ isolated monocytes originated from patients with BP, differentiated into macrophages in presence of BP serum for 7 days and stimulated for 3 h with IL-17 **(C,D)** and IL-23 **(E,F)** (*n* = 6, Wilcoxon's test for paired data was applied; *p ≤ 0.05).

To further clarify the contribution of the IL-17/IL-23 axis to the activation of inflammasome, we first looked for correlation between IL-1β expression and the concentration of IL-17 and IL-23 measured in BFs used to stimulate BP monocyte-derived macrophages in Figure 3. Our results showed a tendency for IL-1β expression to follow the BF concentration of IL-17 but not of IL-23 (*n* = 6; *r* = 0.74, *p* = 0.09 and *r* = −0.30, *p* = 0.55, respectively; Figures 5A,B). We further evaluated the direct capacity of IL-17 and IL-23 to induce the NLPR3-associated inflammasome in BP monocyte-derived macrophages. Priming of the NLRP3 inflammasome by IL-17 was demonstrated in macrophages by a significant increase of the expression of both IL-1β and NLRP3 mRNAs (Figures 5C,D). In contrast, IL-23 significantly induced IL-1β but not NLRP3 mRNA expression, although an increase of NLRP3 mRNA was observed in 5 cases out of 6 (Figures 5E,F). In contrast, inflammasome activation was affected by neither IL-17 nor IL-23, as these cytokines did not enhance IL-1β protein level from LPS primed monocyte-derived macrophages (data not shown).

### IL-1β Induced MMP-9 Secretion in BP Monocyte-Derived Macrophages

**Figure 6:**
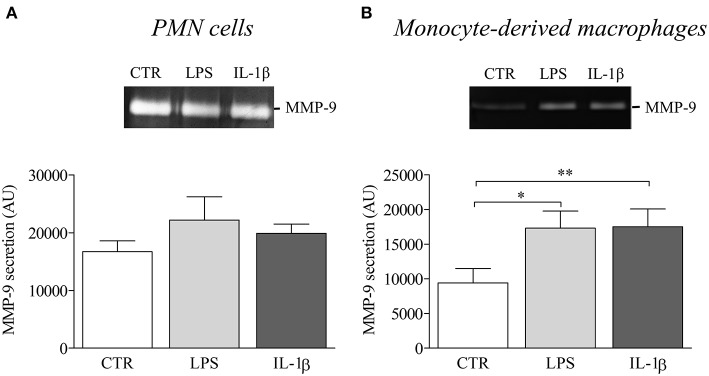
IL-1β induced MMP-9 release in BP serum-differentiated macrophages. MMP-9 release was examined by gel zymography in PMN cells **(A)** and BP monocyte-derived macrophages **(B)** stimulated with LPS or IL-1β for 24 h (*n* = 4; Data are shown as the mean ± SEM; paired *T*-Test was applied for statistical analysis; *p ≤ 0.05; **p < 0.01).

Finally, to investigate the influence of NLRP3-associated IL-1β expression on disease activity of BP, we evaluated the potential role of IL-1β in matrix metalloproteinase-9 (MMP-9) release by macrophages and PMN cells. While PMN-released MMP-9 was not modified by IL-1β stimulation (Figure 6A), we showed a significant increase in MMP-9 secretion by IL-1β-stimulated monocyte-derived macrophages (Figure 6B).

## Discussion

In the context of a precision medicine approach, we defined in this study a subpopulation of patients with BP that displayed resemblances with auto-inflammatory diseases. Indeed, we provided evidence for a differential expression of a functional *in situ* “sterile” inflammasome in patients with BP, biologically characterized by the presence of NLRP3 and IL-1β. Clinically, IL-1β associated inflammasome was related to the presence of erythema and urticarial plaques, which are representative clinical signs of the early inflammatory phase preceding blister formation. Biologically, we identified IL-17 and IL-23 as significant inflammatory mediators for inflammasome priming in BP monocyte-derived macrophages. Finally, the ability of IL-1β to induce macrophage-released MMP-9 supports its participation to the auto-amplification loop of the inflammatory response associated to BP (4).

At the active phase of the disease i.e., at time of diagnosis before treatment, IL-1β was detected at skin lesional site in BF of certain patients with BP but not in all of them, and not in the serum of BP patients. This observation is consistent with previous studies that showed the presence of IL-1β in a small series of BF but not in sera of BP patients (14, 16). Moreover, we also demonstrated a heterogeneous expression of NLRP3 in skin biopsy specimens originated from patients with BP. Corroborating IL-1β findings, some patients strongly expressed NLRP3 both in skin resident cells (keratinocytes, endothelial cells, fibroblasts) and in infiltrated innate immune cells including macrophages and PMN cells, while other showed very low or no expression of NLRP3. Notably, we observed NLRP3-positive PMN cells in the lumen of dermal blood vessels supporting the idea of an additional systemic inflammasome in BP patients that was recently demonstrated with the presence of IL-18 and NLRP3 in peripheral blood mononuclear cells (15). Altogether these results strongly suggest that NLRP3 inflammasome occurred only in certain BP patients, suggesting that either the inflammasome is an epiphenomenon involved in the amplification of the BP-related inflammatory response of the pathophysiological mechanism associated to BP, or that specific BP conditions are required. Accordingly, it was recently shown that expression of specific inflammasome gene module stratifies elderly individuals into two extreme clinical and immunological states (27): those without constitutive expression of IL-1β and the others with constitutive IL-1β that were more susceptible to age-related diseases. In addition, recent studies demonstrated the critical role of skin microbiota in the regulation of the inflammatory response in patients with atopic dermatitis or psoriasis (28). To know if such epiphenomena could have regulatory functions in the inflammasome-related BP pathophysiological mechanisms still needs further investigations and would be of great interest to better classify patients in a future precision medicine approach.

Although future investigations, notably with the use of a control group patients with autoimmune blistering disease sharing autoimmune/inflammation features such as dermatitis herpetiformis, could help to demonstrate whether inflammasome-related mechanisms are specific to BP disease or not, our results showed that such mechanisms are associated with BP activity. Indeed, IL-1β biological activity displayed a positive and significant correlation between the IL-1β levels in BF or the inflammatory ratio IL-1β/IL-1RA and the erythema/urticaria BPDAI subscore, suggesting its involvement in the early inflammatory phase. Notably, no correlation could be demonstrated either between IL-1β/IL-1ra ratio or IL-1β level and the blisters/erosions BPDAI subscore, reinforcing the significance of the “sterile” NLRP3 inflammasome in the early non-bullous urticaria/erythematous phase of the local inflammatory response. Accordingly, no erythema was observed in 2 out 3 patients that did not show NLRP3 expression in skin biopsy specimen in contrast to all NLRP3-highly positive patients that exhibited numerous erythematous plaques (data not shown). Altogether these results highlighted the importance of considering these inflammasome-related mechanisms when initially treating patients with BP. In contrast, no significant relationship could be drawn between the presence of an NLRP3 inflammasome and IL-1β at skin lesional site and the specific auto-immune response defined by anti-BP180 and anti-BP230 serum autoantibodies, suggesting that the inflammasome in BP is rather associated with an auto-inflammatory than an auto-immune process. In our study, neither BF-related IL-1β nor NLRP3 could be identified as a predictive marker of relapse in patients with BP at diagnosis (data not shown).

BF of patients with BP contains all inflammatory molecules classically associated with the priming and the activation of the “sterile” NLRP3-related inflammasome, such as DAMPs (ECM components released from tissue damage), pro-inflammatory cytokines, complement and reactive oxygen species (ROS) (12, 19–22). Indeed, while IL-1β levels did not appeared to be correlated with the blisters/erosions BPDAI subscore, we showed here that BF has the ability to induce priming and activation of an NLRP3-dependent inflammasome in monocyte-derived macrophages through the concomitant regulation of IL-1β and NLRP3 mRNA expression and its increasing effects on IL-1β secretion in LPS-primed macrophage. This was further supported by the use of glibenclamide, a specific inhibitor of NLRP3 inflammasome activation. In line with the positive and significant correlations observed between the concentrations of the pro-inflammatory cytokines IL-17 or IL-23 and IL-1β in BF, we also demonstrated an increased expression of IL-1β mRNA expression in monocyte-derived macrophages primed with BF, especially with BF in which IL-17 concentrations were elevated, and in monocyte-derived macrophages primed with IL-17 or IL-23. These results suggest that IL-1β-dependent inflammation in BP could be at least partly linked to IL-17 and IL-23 but should not overshadow that other molecules contained in BF could also be involved either directly or indirectly in the activation of an inflammasome in BP. Meanwhile, NLRP3 mRNA expression was enhanced in response to IL-17 but not in response to IL-23 suggesting that IL-1β expression could also be regulated in an inflammasome-independent manner as recently highlighted in an experimental caspase-1/11-deficient mouse model of epidermolysis bullosa acquisita (29). We already highlighted discrepancies in the effects of IL-17 and IL-23 in a recent study showing that IL-23 was a more potent inductor of NETosis than IL-17 in BP (30). Release of immunomodulatory cytokines and chemokines during this process could play a role either in favoring the auto-immunity or the auto-inflammatory processes. Besides, although IL-17 induced NLRP3, neither IL-17 nor IL-23 induce the release of IL-1β by macrophage supporting the fact that these cytokines are not directly involved in the generation of the bioactive IL-1β. Thus, these results suggest that other molecules are required to IL-1β production, maybe through the cooperation between different immune cell types or inflammatory processes.

In BP, IL-1β production could involve MMP expression. Indeed MMPs, especially MMP-9, have the ability to cleave pro-IL-1β to generate biologically active IL-1β (31). Noteworthy, both IL-17 and IL-23 were shown to increase MMP-9 production in BP (4, 5). Besides cytokines and MMPs, DAMPs and ROS have also been largely involved in NLRP3 inflammasome activation by notably favoring the NLRP3/ASC complex assembly (20). Investigation of the redox status of BF from patients with BP showed a disturbance of the redox balance toward hyperoxidative status of all BF tested (data not shown), suggesting a potential role of ROS in the activation of the inflammasome in BP. However, ROS being present in all BF do not appear as the limiting factor in the generation of bioactive IL-1β in contrast to the pro-inflammatory cytokine IL-17 that is expressed only in a subpopulation of patients with BP as previously demonstrated (5).

In the present study, IL-1β had the ability to induce MMP-9 secretion in monocyte-derived macrophages but not in PMN cells. *In situ* MMP-9 release could degrade matrix molecules into peptides that could feed back the inflammatory and immune responses (12). Though, together with the pro-inflammatory cytokines IL-17, IL-23 and CXCL10 that we previously demonstrated to induce MMP-9 release in several types of cells (4–6), macrophage-released IL-1β could participate to the auto-amplification loop during the auto-inflammatory response associated with BP. Since IL-1ß plays a pivotal role in the pathogenesis of auto-inflammatory diseases, targeted therapies blocking IL-1 activity by different mechanisms were shown to result in a rapid and sustained reduction of disease severity, including anakinra (IL-1 receptor antagonist), canakinumab (neutralizing monoclonal anti-IL-1β antibody) or rilonacept (soluble decoy receptor) (32). Systemic or superpotent topical corticosteroids are the main effective first-line therapies in BP, but with low selectiveness. They are essentially symptomatic and associated with numerous adverse events. In the context of the precision medicine approach, our results open up new possibilities for therapeutic strategies potentially involving the combination of lower doses of corticosteroids and biotherapies targeting IL-1β pathway or maybe even better targeting pro-inflammatory cytokines such as IL-17 and IL-23 acting upstream of IL-1β to control the auto-inflammatory like process occurring in the early phase of the blister formation in BP.

## Data Availability

All datasets generated for this study are included in the manuscript/supplementary files.

## Author Contributions

SL, PB, and FA designed the study and wrote the manuscript. SL and CM performed experiments. CM, JP, and AD collected clinical data and biological material. SL, CM, JP, PB, and FA analyzed clinical and biological data. All authors critically evaluated the data and approved the final version for publication.

### Conflict of Interest Statement

The authors declare that the research was conducted in the absence of any commercial or financial relationships that could be construed as a potential conflict of interest.
